# Comparison of axial tibial-metatarsal alignment in dogs of varying size with and without medial patellar luxation

**DOI:** 10.3389/fvets.2026.1781687

**Published:** 2026-04-16

**Authors:** Alexandre Silva Gobeti, Justin J. Marrs, Ashton L. Story, Hailey Hilfiker, Derek B. Fox

**Affiliations:** Department of Veterinary Medicine and Surgery, Veterinary Health Center, University of Missouri, Columbia, MO, United States

**Keywords:** canine tibia, patella luxation, proximal tibia metatarsal angle, tibial alignment, tibial torsion

## Abstract

**Objectives:**

To compare tibial torsion angle (TTA) and proximal tibial metatarsal angle (PTMTA) between dogs weighing < 10 kg and dogs weighing ≥ 10 kg with and without medial patellar luxation (MPL) to assess whether transverse plane tibial-metatarsal malalignment is size dependent.

**Study design:**

Retrospective computed tomographic analysis.

**Sample population:**

One hundred and one limbs from 55 client-owned dogs.

**Methods:**

Computed tomographic (CT) scans of pelvic limbs that met inclusion criteria were categorized by dog size (dogs weighing less or over 10 kg) and presence and grade of medial patellar luxation (normal, Grade 2, Grade 3 and Grade 4). A single investigator measured both TTA and PTMTA in each limb. Both TTA and PTMTA were compared between dogs of varying degrees of MPL and between dogs weighing < 10 kg and dogs weighing ≥ 10 kg.

**Results:**

A clear association between increasing TTA and higher grades of MPL was not discerned in dogs weighing < 10 kg. However, dogs weighing < 10 kg did exhibit a trend of worsening tibial-metatarsal malalignment as patellar luxation grade increased with grade 4 MPLs having a significantly larger PTMTA than all other groups. Among dogs weighing ≥ 10 kg, those with grade 3 MPLs possessed a significantly larger TTA compared to all other groups, but dogs with grade 4 MPLs were not different from normal dogs. However, dogs weighing ≥ 10 kg did exhibit a trend of increasing PTMTA values associated with worsening grades of patellar luxation with grade 2 and 4 MPL dogs showing more tibial-metatarsal malalignment than normal dogs. There were differences between dogs weighing < 10 kg and dogs weighing ≥ 10 kg with respect to TTA measurements with dogs < 10 kg showing more tibial torsion in MPL Grades 2 and 4. However, no differences were detected in PTMTA between sizes with any grade of MPL.

**Conclusion:**

Tibial-metatarsal malalignment measurable through an increased PTMTA may be a more predictably associated transverse plane deformity with MPL than tibial torsion alone as measured by the TTA. Both dogs weighing less or over 10 kg appear to exhibit tibial-metatarsal malalignment with increasing severity in cases of higher grades of MPL.

## 1 Introduction

Medial patellar luxation (MPL) is a common cause of rear limb lameness in the dog. Patellar laxity is frequently accompanied by pelvic limb malalignment (1). The correlation between axial malalignment of the tibia and the tibial-metatarsal relationship and MPL has been the subject of numerous studies (1–5). Assessment of tibial torsion and tibial-metatarsal rotation in the transverse plane have been investigated in detail via computed tomography (CT). These characteristics can be quantified, in part, through the measurement of the tibial torsion angle (TTA) (6) and proximal tibial metatarsal angle (PTMTA) (3, 4). Examination of these parameters has demonstrated that with high grade medial luxation of the patella, the proximal tibia exhibits internal torsion with respect to the distal tibia, and the metatarsals exhibit external rotation with respect to the tibia (1, 3). It is believed that these changes may occur during skeletal development to compensate for a fixed, internally rotated tibia that occurs during continuous medial luxation of the patella to normalize foot position (1). While tibial torsion alone, quantifiable via measuring the TTA on CT, is a major component of tibial-metatarsal alignment, drawbacks with its use have been noted including lacking consideration of the pes, difficulty identifying some landmarks in dogs weighing < 10 kg (5, 7), low interobserver reliability (4) and the absence of palpable correlates on physical examination (3). The PTMTA represents a composite measurement, assessing the entirety of the extremity in the transverse plane distal to the femur. This measurement therefore reflects the relationship of the proximal tibia to the pes, includes both tibial torsion and intratarsal rotation and can be visibly confirmed during gross examination (2–4). Additional research has demonstrated that the PTMTA can be reliably measured with excellent repeatability and reproducibility (4). A recent study has documented that higher values of the PTMTA are directly correlated with higher grades of patellar luxation (3). Most studies that have assessed axial tibial and tibial-metatarsal malalignment in dogs with MPL have only examined dogs weighing < 10 kg (1–3, 5). It is still incompletely understood if this malalignment is equally as significant in dogs weighing ≥ 10 kg that have been diagnosed with MPL. Therefore, the objectives of this study were to measure the TTA and PTMTA in dogs with varying grades of MPL and compare these values between dogs weighing < 10 kg and dogs weighing ≥ 10 kg. We hypothesized that higher grades of MPL would demonstrate significantly greater axial malalignment consistent with previous reports, and that no differences would be seen between dogs weighing < 10 kg and dogs weighing ≥ 10 kg.

## 2 Materials and methods

### 2.1 Case selection

Medical records from the University of Missouri’s Veterinary Health Center database were searched for patients that received a CT analysis of one or both pelvic limbs between 2017 and 2024. Patient data collection included signalment, body weight, presenting complaint, physical and orthopedic examination findings, presumptive diagnoses, all diagnostic images acquired and the associated imaging reports. Dogs were routinely positioned for a CT scan by a radiologist in sternal recumbency on a foam V-trough with a square foam between the pelvic region, to keep the spine aligned, and the rear limbs in the most anatomical position to a normal standing tibial tarsal angle. Inclusion criteria for CT scans to be considered for review were as follows: Images containing the entire rear limb including pelvis, femur, tibia and pes were included. Patients that present with a tibiotarsal flexion angle of 108.0° to 169.0° on the sagittal plane imaging reconstruction analysis were included. Tibiotarsal flexion angle, measured between the long axis of the tibia and the long axis of the metatarsus on the sagittal plane was performed in each scan. The standing tibial tarsal angle has been previously described as 135.0–145.0° in canines (8), demonstrated in a prior a canine standing CT study with a tarsal joint/extension angle been 110.9–128.7° (1) and in a goniometer study been described as an extension angle of 162.0–166.0° (9). Exclusion criteria included dogs diagnosed with lateral patellar luxation, fractures or malunions of any bone of the pelvic limb, inadequately positioned limbs, and patients that did not meet the tibiotarsal flexion angle stablished above. Patients were divided into one of four groups based on the status of their medial patellar luxation using the classification established by Roush (10): normal (no luxation), grade 2, grade 3, and grade 4. Patients were then separated by weight into two groups: dogs weighing less than 10 kg and dogs equal to and greater than 10 kg.

### 2.2 Imaging acquisition and assessment

All CT scans were acquired using a 640-slice helical scanner (Canon Aquilion One, Canon Medical Systems, United States, Tustin, California). Slices from the proximal and distal tibial segments were examined in a multiplanar reconstruction (MPR) for the presence of specific landmarks to allow determination of TTA and captured for analysis as previously described (6). DICOM (Digital Imaging and Communications in Medicine) files were collected for the CT scans of each limb. Three-dimensional (3D) volumetric reconstructions of the limbs from the level of the proximal tibia distally were performed using Vitrea Advanced Visualization (version 7; Canon Medical Systems, Tustin, CA, United States). Proximal and distal tibial 2D slices (defined in MPR) with the specific landmarks (6), plus static three-dimensional (3D) views of the volumetric reconstructions in the sagittal, frontal and transverse (axial) planes, were exported to a dedicated orthopedic planning software VPOP PRO (version 3.0; VetSOS Education Ltd., Shrewsbury, United Kingdom) upon which all measurements were made by a single investigator (ASG). Sagittal plane images of the reconstructions were used to confirm tibiotarsal flexion angle (angle between the long axis of the tibia and the long axis of the metatarsus). TTA was measured from tibial cross-sectional slices according to the methodology described by Aper et al. (6) and PTMTA was measured from three-dimensional (3D) volumetric reconstructions of the tibia, tarsus and pes according to the measuring methodology described by Isono et al. (3). The means and standard deviations of both angles were recorded.

### 2.3 Statistical analysis

All statistical analyses were performed using the software RStudio (Version: 2025.09.2 + 418, Posit Software, PBC, Boston, MA, United States). Normality of data was assessed using Shapiro–Wilk test. Given the potential deviations from normality and unequal variances, a two-sided Welch’s two sample *t*-test was performed, to test for significant differences in either TTA or PTMTA, between paired combinations of MPL groups (normal, Grade 2, Grade 3 and Grade 4). A two-sided Welch’s two sample *t*-test was also performed to test for significant differences in either TTA or PTMTA between dogs weighing < 10 kg and dogs weighing ≥ 10 kg across MPL grades. Statistical significance was set at *p* < 0.05.

## 3 Results

CT images from 101 pelvic limbs (55 dogs) met the inclusion criteria for this study and were evaluated. The mean tibiotarsal angle in the population was (154.0° ± 11.5°, 108.0° – 169.0°). In normal dogs weighing < 10 kg the mean tibiotarsal angle in the population was (147.0° ± 15.5°, 108.0° – 158.0°). In the MPL grade 2/4 group on dogs weighing < 10 kg the mean tibiotarsal angle in the population was (164.0° ± 3.1°, 162.1° – 166.6°). In the MPL grade 3/4 group on dogs weighing < 10 kg the mean tibiotarsal angle in the population was (151.3° ± 17.7°, 113° – 165.8°). In the MPL grade 4/4 group on dogs weighing < 10 kg the mean tibiotarsal angle in the population was (151.7° ± 16°, 117.1° – 167.4°). In the normal group on dogs weighing ≥ 10 kg the mean tibiotarsal angle in the population was (153.0° ± 9.1°, 123.9° – 167.4°). In the MPL grade 2/4 group on dogs weighing ≥ 10 kg the mean tibiotarsal angle in the population was (155.5° ± 6.9°, 148.0° – 165.2°). In the MPL grade 3/4 group on dogs weighing ≥ 10 kg the mean tibiotarsal angle in the population (159.0° ± 5.3°, 150.0° – 167.9°). In the MPL grade 4/4 group on dogs weighing ≥ 10 kg the mean tibiotarsal angle in the population was (157.0° ± 7.4°, 146.0° – 169.0°).

The mean age at presentation was 3.1 years (range: 7 months to 9 years) and the mean body weight was 24.1 kg (range: 2.8 kgs – 75 kg). There were castrated male (*n* = 21), intact male (*n* = 6), spayed female (*n* = 23) and intact female (*n* = 5) dogs. Breeds represented included mixed-breed (*n* = 13), Labrador Retriever (*n* = 5), German Shepherd Dog (*n* = 4), Yorkshire Terrier (*n* = 4), English Bulldog (*n* = 3), American Staffordshire Terrier (*n* = 3), Chihuahua (*n* = 3), Great Pyrenees (*n* = 2), Dachshund (*n* = 2), Shiba Inu (*n* = 2), Cavalier King Charles Spaniel (*n* = 2), Pomeranian (*n* = 2), American Bully (*n* = 1), Australian Shepherd (*n* = 1), Belgian Malinois (*n* = 1), Mastiff (*n* = 1), Golden Retriever (*n* = 1), German Shorthair Pointer (*n* = 1), Great Dane (*n* = 1), Bernese Mountain Dog (*n* = 1), and Swiss Mountain Dog (*n* = 1). Forty-nine right limbs and 52 left limbs were examined. Forty-four limbs did not have patellar luxation on orthopedic examination and were categorized as “normal.” Seven limbs had grade 2 MPL, 23 limbs had grade 3 MPL, and 27 limbs had grade 4 MPL. Thirty-three limbs were from dogs weighing less than 10 kg and 68 limbs were from dogs weighing equal to or more than 10 kg.

Among dogs under 10 kg, the TTA was largely unaffected by MPL grade. Dogs with grade 2 MPL demonstrated a significantly higher TTA than normal dogs, however, were underrepresented in the population (Table 1). When examining PTMTA in dogs weighing < 10 kg, those with grade 4 MPL demonstrated significantly more malalignment than all other groups whereas no differences were detected between normal, grade 2 and grade 3 MPL-affected dogs (Table 2).

**Table 1:** Tibial torsion angles (TTA)^6^ among dogs weighing < 10 kg and dogs weighing ≥ 10 kg diagnosed with varying grades of medial patellar luxation (MPL).

MPL grade	Size of dog		*p*-value for < 10 kg vs. ≥ 10 kg dog comparisons
	Weighing < 10 kg	Weighing ≥ 10 kg	
Normal	9.92° ± 8.76° (*n* = 11)^b^	10.45° ± 8.17° (*n* = 33)	0.86
Grade 2	19.95° ± 1.06° (*n* = 2)^a^	11.14° ± 4.90° (*n* = 5)	0.01^*^
Grade 3	13.82° ± 15.31° (*n* = 6)	13.39° ± 7.19° (*n* = 17)^d^	0.95
Grade 4	14.82° ± 2.13° (*n* = 14)	5.69° ± 8.69° (n = 13)^c^	0.01^*^

Tibial torsion angles (TTA) listed as the mean ± standard deviation with the sample size (n = number of limbs) for each group written in parentheses. Within the same column of dog sizes, lowercase superscript letters indicate significant difference between grades *p* < 0.05; ^a^vs. Normal; ^b^vs. Grade 2; ^c^vs. Grade 3; ^d^vs. Grade 4. Within the same row of MPL grade, significant differences between dog sizes indicated with an asterisk (*p* < 0.05).

**Table 2:** Proximal tibial metatarsal angle (PTMTA)^3^ among dogs weighing < 10 kg and dogs weighing ≥ 10 kg diagnosed with varying grades of medial patellar luxation (MPL).

MPL grade	Size of dog		*p*-value for < 10 kg vs. ≥ 10 kg dog comparisons
	Weighing < 10 kg	Weighing ≥ 10 kg	
Normal	4.61° ± 2.53° (*n* = 11)^d^	8.05° ± 6.64° (*n* = 33)^b,d^	0.02^*^
Grade 2	6.9° ± 5.37° (*n* = 2)^d^	18.08° ± 5.11° (*n* = 5)^a^	0.14
Grade 3	9.08° ± 4.44° (n = 6)^d^	11.52° ± 7.28° (n = 17)^d^	0.61
Grade 4	25.97° ± 16.27° (*n* = 14)^abc^	22.37° ± 11.54° (*n* = 13)^a,c^	0.56

Proximal tibial metatarsal angle (PTMTA) listed as the mean ± standard deviation with the sample size (n = number of limbs) for each group written in parentheses. Within the same column of dog sizes, lowercase superscript letters indicate significant difference between grades *p* < 0.05; ^a^vs. Normal; ^b^vs. Grade 2; ^c^vs. Grade 3; ^d^vs. Grade 4. Within the same row of MPL grade, significant differences between dog sizes indicated with an asterisk (*p* < 0.05).

Among dogs over 10 kg, the only difference detected in TTA was between dogs with grade 3 and 4 MPL with the latter showing significantly less torsion (Table 1). Conversely, dogs with grade 4 MPL showed a higher PTMTA compared to dogs with grade 3 MPL (Table 2). Examination of PTMTA also revealed a trend of increasing tibial-metatarsal malalignment with increasing luxation grade, as dogs with grade 2 and 4 MPL had significantly more external rotation than normal dogs.

When comparing TTA measurements between dog sizes, differences were detected in both the grade 2 and grade 4 MPL groups. In both situations, TTA was greater in the dogs weighing < 10 kg (Table 1). When comparing PTMTA, the only difference detected between size cohorts was in normal dogs where dogs weighing ≥ 10 kg demonstrated a higher PTMTA than dogs weighing < 10 kg (Table 2).

## 4 Discussion

Based on our study, our results provide a partial validation of our hypothesis. While the overall trend of malalignment worsening with higher grades of MPL was confirmed, the specific measurement tools (TTA vs. PTMTA) yielded inconsistent results, and some differences were observed between size groups that our hypothesis did not initially predict.

The results suggest that tibial–metatarsal malalignment may be more consistently associated with increasing severity of MPL than tibial torsion alone. This trend was evident in both dogs weighing < 10 kg and dogs weighing ≥ 10 kg, although differences in the magnitude of the malalignment differed between size groups.

In dogs weighing less than 10 kg, increasing MPL grade was not associated with a clear or progressive increase in tibial torsion. While dogs with grade 2 MPL demonstrated a significantly higher TTA than normal dogs, this finding should be interpreted cautiously due to the very small number of limbs in this subgroup and the possibility of a type I statistical error. In contrast, PTMTA values demonstrated a more predictable relationship with MPL severity in dogs weighing < 10 kg, with grade 4 MPL limbs exhibiting significantly greater tibial–metatarsal malalignment compared with all other groups. These findings suggest that, in dogs weighing < 10 kg, malalignment of the distal extremity associated with MPL may be more consistently associated with tibial-metatarsal rotation, including contributions from the pes and tarsus, rather than tibial torsion alone.

Among dogs weighing 10 kg or more, the relationship between MPL grade and TTA was similarly inconsistent. Only dogs with grade 3 MPL demonstrated significantly greater tibial torsion compared to those with grade 4 MPL, while no differences were identified between grade 4 MPL limbs and normal limbs. The lack of significant differences in TTA by MPL grade relative to PTMTA by MPL grade suggests that transverse plane deformities of the distal extremity in dogs weighing ≥ 10 kg with MPL cannot be characterized by tibial torsion alone. In contrast, PTMTA measurements in dogs weighing ≥ 10 kg demonstrated a trend toward increasing tibial–metatarsal malalignment with worsening MPL grade. Dogs affected by grade 2 and grade 4 MPL exhibited significantly greater malalignment than normal dogs. These findings further support the concept that assessing the composite tibial–metatarsal alignment may better reflect the transverse plane abnormalities seen in the distal extremity associated with MPL across a wider range of body sizes.

When comparing dogs weighing < 10 kg and dogs weighing ≥ 10 kg directly, differences in TTA were detected in the grade 2 and grade 4 MPL groups, with dogs weighing < 10 kg exhibiting greater tibial torsion. This finding may reflect developmental or biomechanical differences between dogs weighing < 10 kg and dogs weighing ≥ 10 kg, including differences in physeal growth patterns or breed-related conformation. However, despite these differences in TTA, PTMTA values did not differ between size cohorts within any MPL grade, suggesting that the overall degree of tibial–metatarsal malalignment associated with MPL is comparable between dogs weighing < 10 kg and dogs weighing ≥ 10 kg and worsens with higher grades of MPL. This finding suggests that whereas tibial torsion is inconsistently exhibited with increasing severity of MPL in dogs weighing < 10 kg and dogs weighing ≥ 10 kg, tibial-metatarsal malalignment becomes more exaggerated with worsening MPLs in all sizes of dogs.

The findings of this study differ from previous work which demonstrated association of worsening tibial torsion as measured by TTA in dogs weighing < 10 kg with increasing severity of MPL grade (2, 3, 5). An important distinction with several of those studies, however, is the examination of only a single breed in each report; Toy poodles (2) and Yorkshire terriers (5). The work by Isono et al. focused on a mixed population of dogs weighing < 10 kg, which Toy poodles were overrepresented at 21% (3). We speculate that the greater diversity of breeds examined in this study (in both dogs weighing < 10 kg and dogs weighing ≥ 10 kg subgroups) may have resulted in a larger variation in what may be conformation-dependent measurements. While the study used a 10 kg cutoff for grouping, future studies with larger cohorts might benefit from a more granular classification aligned with the established veterinary size categories. To date, no comparative studies have examined breed differences on tibial-metatarsal malalignment with patellar luxation. Another possibility of why these results differ from previous studies could be related to the challenges of reliably measuring TTA in dogs weighing < 10 kg and dogs weighing ≥ 10 kg (4, 5). Although the technique for measuring TTA was first established in normal larger breeds (6), recent studies have shown that reproducibility of the technique in a mixed population of dogs with patellar luxation is lacking (4). While the TTA and PTMTA measurements are inherently two-dimensional, the use of 3D volumetric CT reconstructions facilitates accurate and repeatable identification of anatomical landmarks and the generation of standardized measurement planes, thereby enhancing the reliability of these 2D measurements. Our findings regarding the effect of higher grades of MPL on PTMTA are consistent with previous reports (3). Since PTMTA is a tibiotarsal angle-dependent measurement, the variability in tarsal angle across our patients is a confounding factor that must be considered when interpreting the results.

This study has several limitations. The retrospective nature resulted in limited control over case selection and identical limb positioning relying on the standardized radiologist positioning of the patients. Further, the distribution of MPL grades within size groups particularly Grade 2 MPL and Grade 3 MPL in dogs weighing < 10 kg, was uneven resulting in underrepresentation of the subgroups that could have resulted in statistical error. Given the retrospective nature of this study, an *a priori* power analysis was not performed. Analyzing a mixed population of dogs likely led to greater variation in data related to diversity of breed conformation. Measurements were performed by a single investigator, precluding assessment of interobserver variability. While a standardized positioning protocol was followed, a brace could further enhance consistency, although the use of a positioner brace is not routine in orthopedic CT’s. Another limitation is the range of the tibiotarsal angle evaluated, the PTMTA could’ve been affected by the different tibiotarsal angles. Further studies should evaluate and compare the degree of changes on the PTMTA with the different tibiotarsal angles in the same patient. Despite these limitations, standardized CT acquisition protocols and consistent measurement techniques based on well-founded research were used to mitigate potential sources of error, however, these do not negate the impact of limited sample sizes in certain subgroups.

The observed sample sizes, particularly for certain subgroups (e.g., Grade 2 MPL in dogs weighing < 10 kg), are acknowledged as a limitation, potentially affecting the statistical power to detect significant differences. Future prospective studies with larger and more evenly distributed populations are warranted to further clarify the relationship between transverse plane malalignment and MPL severity, particularly in dogs weighing ≥ 10 kg. Clinically, these findings suggest that reliance on tibial torsion measurements alone may underestimate the degree of transverse plane malalignment in dogs with MPL, particularly in higher grades. Assessment of tibial–metatarsal alignment may therefore be valuable when evaluating dogs of all sizes with MPL, especially when considering surgical planning and correction. Recognition that dogs weighing ≥ 10 kg exhibit clinically relevant tibial–metatarsal malalignment should influence preoperative imaging strategies and help guide surgical decision-making.

In conclusion, increased tibial–metatarsal malalignment, as measured by the PTMTA, appears to be more consistently associated with increasing severity of medial patellar luxation than tibial torsion alone. Both dogs weighing < 10 kg and dogs weighing ≥ 10 kg demonstrate transverse plane malalignment with advancing MPL grade, supporting the clinical relevance of evaluating the tibial–metatarsal relationship in dogs of all sizes affected by MPL.

## Data Availability

The original contributions presented in the study are included in the article/supplementary material, further inquiries can be directed to the corresponding author.
